# Supplementary material for: Tyrosol blocks E. coli anaerobic biofilm formation via YbfA and FNR to increase antibiotic susceptibility
Source: Nat Commun. 2024 Jul 6;15:5683. doi: 10.1038/s41467-024-50116-3 (PMC11227560; doi:10.1038/s41467-024-50116-3)
Supplement: Supplementary file 1 — Supplementary Information [file 41467_2024_50116_MOESM1_ESM.docx]

**Supporting Information**

**Tyrosol blocks *E. coli* anaerobic biofilm formation via YbfA and FNR to increase antibiotic susceptibility**

**Table S1**. Primers used for quantitative RT-PCR in *E. coli*

| Gene and primer type | PCR primer sequence (5' to 3') |
| --- | --- |
| nirB |  |
| Forward | CGTTTACCTCGACGAAAGTA |
| Reverse | TTTTCCGGCAGTTCGATAGC |
| nrfA |  |
| Forward | ACGGCAAAAACAAAGCGGTT |
| Reverse | TTTCATATTCCGGGTGCTGC |
| narG |  |
| Forward | AAACCCAGCAGACTGACTAT |
| Reverse | CATTTTCATCAGGCGTTTGC |
| napA |  |
| Forward | AAGCCATCAAATGGGATAAA |
| Reverse | GGAAATAGCCCTTAATGCAG |
| Fnr |  |
| Forward | TTTGACGCCATCGGCAGC |
| Reverse | TTCACCGCTCATCAGACGCA |
| narZ |  |
| Forward | TACAGCGACATGCCGATGCT |
| Reverse | ACCTGCGGTATTAACTGCTA |
| Hmp |  |
| Forward | CGCGAGGCGGAAATCTATAA |
| Reverse | GTATTCTGCCACTGCGCC |
| yfeR |  |
| Forward | CCAGACCATTTCCGCGCA |
| Reverse | TCGATGACGATGCCAAAATC |
| fliA |  |
| Forward | GGCACAGGCAATAGGGCA |
| Reverse | CGCCACTCATCGTAGGAGA |
| flgC |  |
| Forward | GTTAACGCTGCACCAGGT |
| Reverse | TCCGACAACATCAACGTTCG |
| flgN |  |
| Forward | GGCGACGCTGGATTACCT |
| Reverse | TGTCCTTCCAGTAACCAGCC |
| hycF |  |
| Forward | CCCGTCAAACGCCTTAAC |
| Reverse | ACCGCCAGTTCGTACTCT |
| hycI |  |
| Forward | TCGTCGCTATCCGTGAAC |
| Reverse | GGTAATTCAACGGCATGTTA |
| hyaA |  |
| Forward | GATGAGCGCCATCATTAC |
| Reverse | CAACTCTGGACGAACTCT |
| rpoD |  |
| Forward | AGGAAGATCTGGACGATGAC |
| Reverse | CTTTGATGGTGTCACGCGTT |

**Table S2**. Primers used for overexpression plasmid construction

| Gene and primer type | PCR primer sequence (5' to 3') |
| --- | --- |
| ybfA |  |
| Forward | ATGGAACTCTACAGAGAATATCCTG |
| Reverse | TCAATAAAAATCACCAGTTGCCTTT |
| napA |  |
| Forward | ATGAAACTCAGTCGTCGTAGCTTT |
| Reverse | TTACACCTTCTCCAGTTTGACCG |
| yfeR |  |
| Forward | ATGAATTATTCTTTAAAACAATTAA |
| Reverse | CTATATCTGATACAACGGAT |
| fnr |  |
| Forward | ATGATCCCGGAAAAGCGAATTATA |
| Reverse | TCAGGCAACGTTACGCGTAT |
| pnapA |  |
| Forward | CGACGCGCCGAGCGGTCA |
| Reverse | CGCGGTCAAACTGGAGAAGGTGTAA |
| pyfeR |  |
| Forward | CCAGCAACACCACCGTGATC |
| Reverse | CTATATCTGATACAACGGATCCCCT |
| pfnr |  |
| Forward | TTACCCTTAACAACTTAAGGGTTTT |
| Reverse | TCAGGCAACGTTACGCGTAT |

**Table S3**. Primers used for cloning of recombinant proteins

| Gene and primer type | PCR primer sequence (5' to 3') |
| --- | --- |
| ybfA |  |
| Forward | AAAAAACATATGATGGAACTCTACAGAGA |
| Reverse | AAAAAAAAGCTTTCAATAAAAATCACCAGT |
| yfeR |  |
| Forward | AAAAAACATATGATGAATTATTCTTTAA |
| Reverse | AAAAAAAAGCTT CTATATCTGATACAA |

**Table S4**. Primers used for site-directed mutagenesis

| YbfA variants and primer type | PCR primer sequence (5' to 3') |
| --- | --- |
| A19G |  |
| Forward | TTACGCCGTACTTATGGGGTTGCAGCGGGCGTT |
| Reverse | AACGCCCGCTGCAACCCCATAAGTACGGCGTAA |
| V20G |  |
| Forward | CGCCGTACTTATGCGGGTGCAGCGGGCGTTCTG |
| Reverse | CAGAACGCCCGCTGCACCCGCATAAGTACGGCG |
| L43G |  |
| Forward | CGCTTTTACAGCTACGGGCATCGCGTCTGGTCG |
| Reverse | CGACCAGACGCGATGCCCGTAGCTGTAAAAGCG |
| W47A |  |
| Forward | TACCTGCATCGCGTCGCGAAAACCAGCGATAAA |
| Reverse | TTTATCGCTGGTTTTCGCGACGCGATGCAGGTA |
| Y40A |  |
| Forward | GACCGCGCCCGCTTTGCCAGCTACCTGCATCGC |
| Reverse | GCGATGCAGGTAGCTGGCAAAGCGGGCGCGGTC |
| H44A |  |
| Forward | GCTTTTACAGCTACCTGGCTCGCGTCTGGTCGAAAAC |
| Reverse | GTTTTCGACCAGACGCGAGCCAGGTAGCTGTAAAAGC |

**Fig. S1**

**
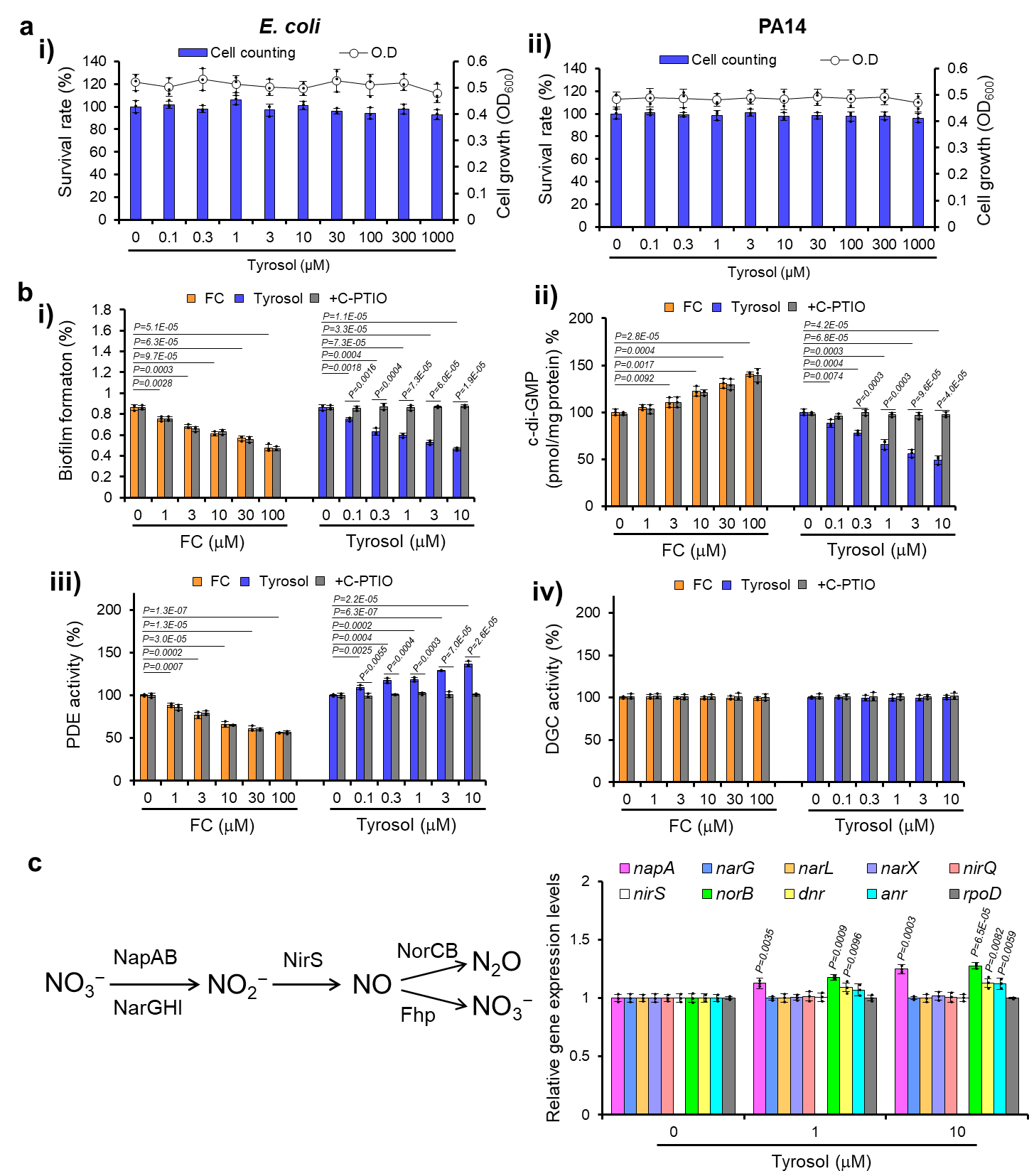
**

**Fig. S1**. Tyrosol inhibits *P. aeruginosa* biofilm formation and reduces cellular c-di-GMP levels by stimulating PDE activity, and these effects are blocked by the NO scavenger C-PTIO.

(**a**) Effects of tyrosol on bacterial growth. *E. coli* BW25113 (**i**) and *P. aeruginosa* PA14 (**ii**) biofilms were formed in the presence of various concentrations of tyrosol for 18 and 9 h, respectively, followed by the measurement of planktonic cells at 600 nm. After the biofilms were dissociated from the wells by gentle sonication and planktonic cells were harvested by centrifugation, the number of viable cells in the planktonic and biofilms cells was counted in each culture. (**b**) Biofilm formation, c-di-GMP levels, PDE, and DGC activities in *P. aeruginosa* PA14 formed in the presence of various concentrations of tyrosol or furanone C-30 (FC) as a positive control and in the absence or presence of C-PTIO (2-(4-carboxyphenyl)-4,4,5,5-tetramethylimidazoline-1-oxyl-3-oxide) for 9 h. The experiment shown is representative of three independent experiments, and the mean ± SD of three independent biological replicates are displayed as each bar. *P*-values were determined using a two-sided Student’s *t*-test. (**c**) Expression of the denitrification genes and related genes in *P. aeruginosa* biofilms formed in the presence of different tyrosol concentrations for 12 h as assessed by RT-qPCR. The experiment shown is representative of three independent experiments, and the mean ± SD of three independent biological replicates are displayed in each bar. *P*-values were determined using a two-sided Student’s *t*-test and data were compared with untreated cells. Source data are provided as a Source Data file.

**Fig. S2**

**
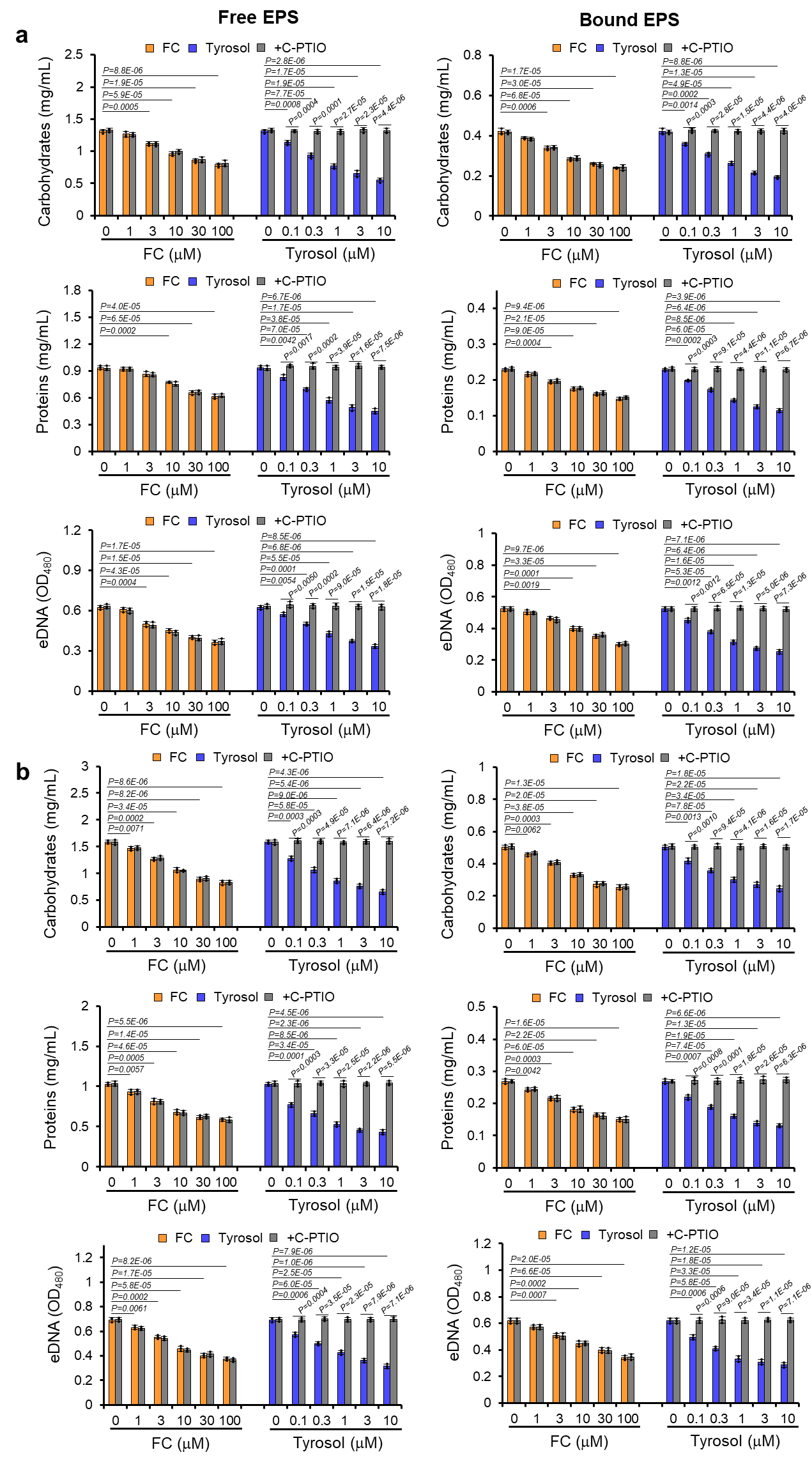
**

**Fig. S2**. Tyrosol inhibits the production of extracellular polymer substances (EPS) in *E. coli* and *P. aeruginosa* biofilm formation, and these effects are blocked by the NO scavenger C-PTIO. Production of free and bound EPS in biofilm formation of *E. coli* BW25113 (**a**) and PA14 (**b**) in the presence of different concentrations of tyrosol or furanone C-30 (FC) as a positive control, and in the absence or presence of C-PTIO (2-(4-carboxyphenyl)-4,4,5,5-tetramethylimidazoline-1-oxyl-3-oxide), for 18 h. Free and bound EPS were extracted from the medium and the attached biofilms, respectively, and three components of EPS (extracellular carbohydrates, proteins, and extracellular DNA (eDNA)) were then assessed. In **a** and **b**, the experiment shown is representative of two independent experiments, and the mean ± SD of three independent biological replicates are displayed in each bar. *P*-values were determined using a two-sided Student’s *t*-test. Source data are provided as a Source Data file.

**Fig. S3**

**
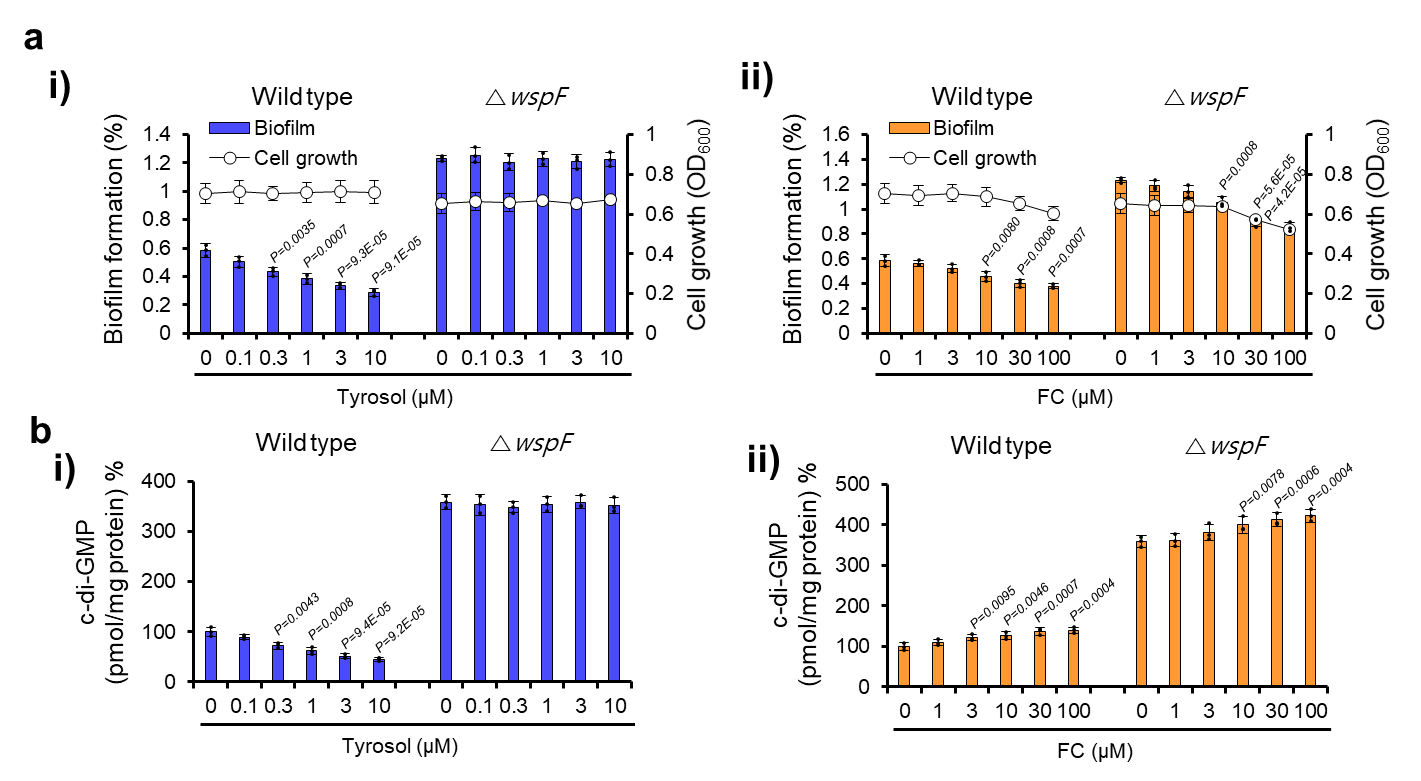
Fig. S3.** Tyrosol inhibits *P. aeruginosa* biofilm formation by lowering cellular c-di-GMP levels. (**a**) Comparison of biofilm formation by the *P. aeruginosa* wspF mutant compared to wild-type PA14 cells cultured with different concentrations of tyrosol (**i**) or furanone C-30 (FC) (**ii**) for 9 h. (**b**) Cellular c-di-GMP levels in the biofilm cells of *P. aeruginosa* wspF mutant compared to wild-type PA14 cells cultured with different concentrations of tyrosol (**i**) or FC (**ii**) for 9 h. After the biofilms were dissociated from the wells by gentle sonication, cellular c-di-GMP was extracted from the biofilm cells, measured, and normalized by total proteins. In **a** and **b**, the experiment shown is representative of two independent experiments, and the mean ± SD of three independent biological replicates are displayed in each bar. *P*-values were determined using a two-sided Student’s *t*-test and data were compared with untreated cells. Source data are provided as a Source Data file.

**Fig. S4**

**
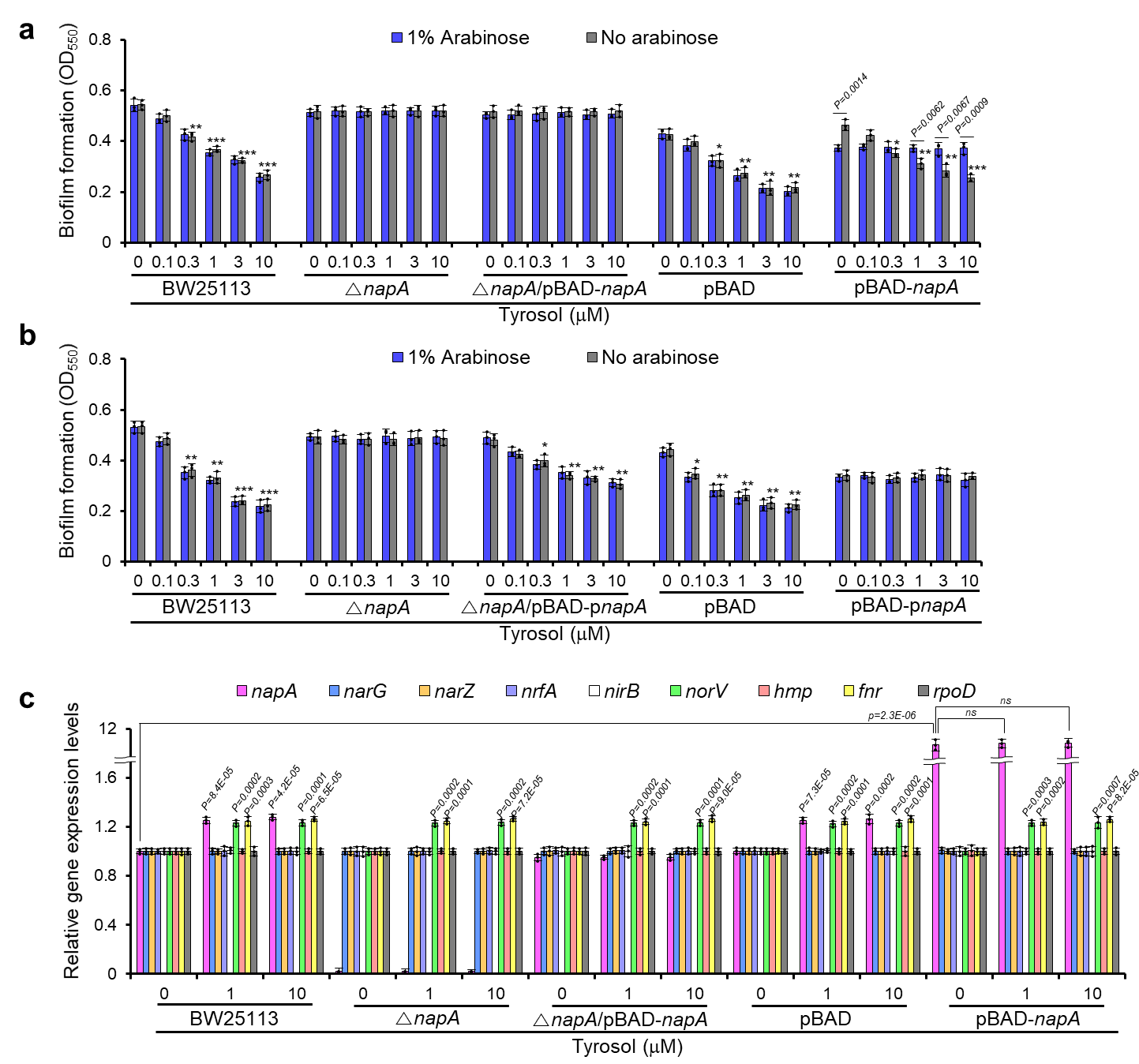
**

**Fig. S4**. Tyrosol shows antibiofilm activity by upregulating *napA* transcription in *E. coli*.

(**a**, **b**) Tyrosol requires the promoter region of *napA* for antibiofilm activity. Biofilm formation in *E. coli* BW25113, an nitrate reductase mutant (△*napA*), the △*napA* strain complemented with *napA* (**a**) or p*napA* (*napA* with an promoter region) (**b**) (Δ*napA* (*pBAD-*△*napA*) or Δ*napA* (*pBAD-*△p*napA*), *E. coli* BW25113 containing only a vector (pBAD), and a *napA* (**a**) or p*napA* (**b**) -overexpressing *E. coli* BW25113 strain (pBAD-*napA* or pBAD-p*napA*) cultured in the presence of different concentrations of tyrosol and in the absence or presence of arabinose. (**c**) Tyrosol did not increase *napA* transcription without its promoter region. Expression of the NAR-encoding genes and related genes in biofilm cultures of BW25113, the Δ *napA* strain, the Δ*napA* (*pBAD-*△*napA*) strain, the pBAD strain, and the pBAD-*napA* strain cultured in the presence of different concentrations of tyrosol and in presence of arabinose for 18 h as assessed by RT-qPCR. In **a** and **b**, the experiment shown is representative of three independent experiments performed, and the mean ± SD of three independent biological replicates are displayed in each bar. *P*-values were determined using a two-sided Student’s *t*-test. *, **, and *** indicate *P* < 0.01, *P* < 0.001, and *P* < 0.0001, respectively, compared to untreated cells. . In **c**, the experiment shown is representative of three independent experiments performed, and the mean ± SD of three independent biological replicates are displayed in each bar. *P*-values were determined using a two-sided Student’s *t*-test and, unless otherwise indicated, data were compared with untreated cells. ns indicates not significant. Source data are provided as a Source Data file.

**Fig. S5**

**
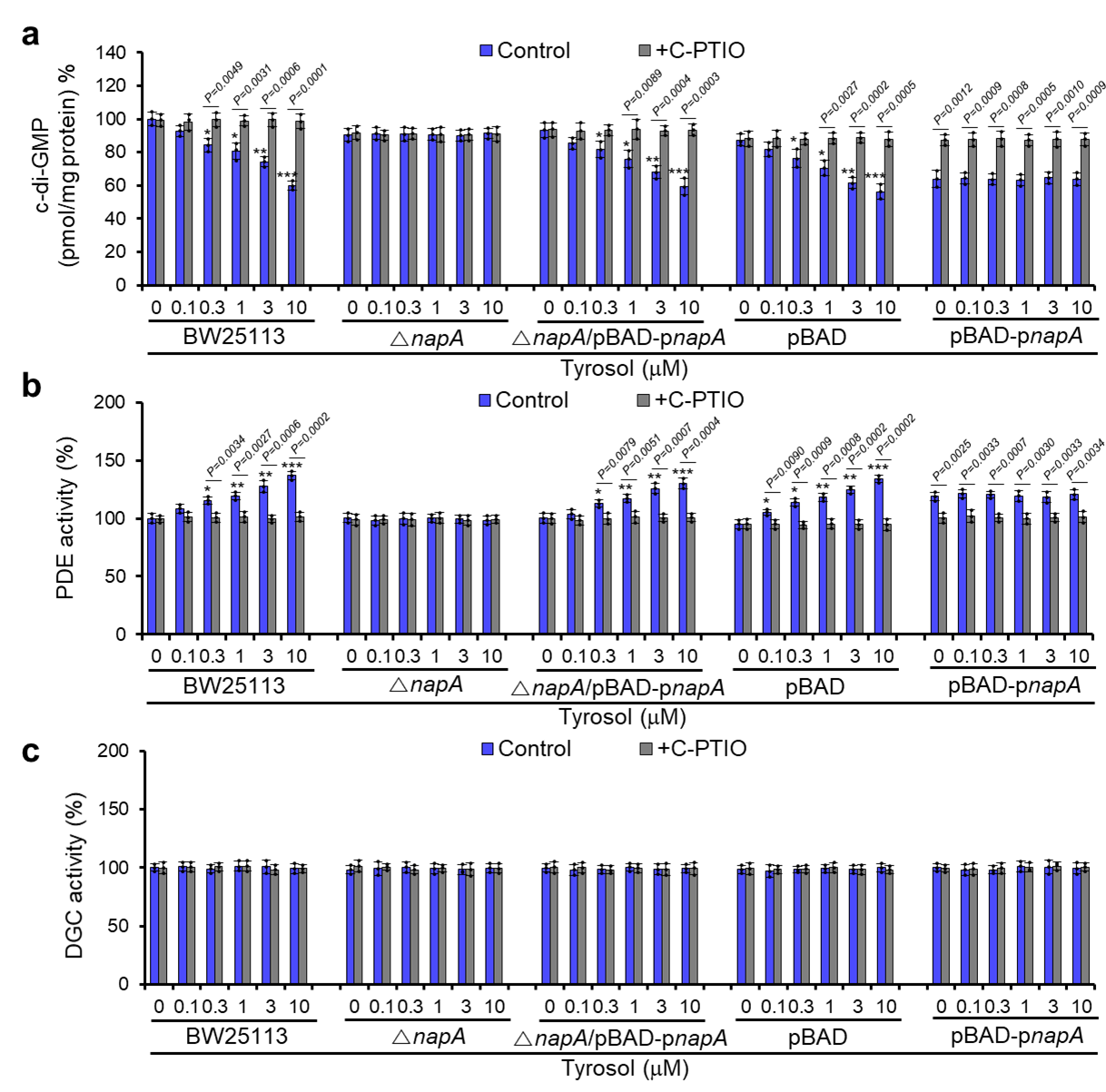
**

**Fig. S5**. Decreases in c-di-GMP levels and elevation in PDE activity by tyrosol requires the periplasmic nitrate reductase (NAR) *napA*. (**a**, **b**, **c**) Effects of tyrosol on cellular c-di-GMP levels (**a**), PDE (**b**), and DGC (**c**) activity in *E. coli* BW25113, an nitrate reductase mutant (△*napA*), the △*napA* strain complemented with p*napA* (*napA* with an promoter region) *(*Δ*napA* (*pBAD-*△p*napA*)), *E. coli* BW25113 containing only a vector (pBAD), and a p*napA* -overexpressing *E. coli* BW25113 strain (pBAD-p*napA*). Cellular c-di-GMP levels in biofilm cultures of each *E. coli* strains grown with different tyrosol of concentrations in the absence or presence of C-PTIO (2-(4-carboxyphenyl)-4,4,5,5-tetramethylimidazoline-1-oxyl-3-oxide) for 18 h. After the biofilms were dissociated from the wells by gentle sonication, cellular c-di-GMP was extracted from the biofilm cells, measured, and normalized to the total proteins. PDE and DGC activity in each *E. coli* strains cultured with different concentrations of tyrosol in the absence or presence of C-PTIO for 24 h. In **a** and **b**, the experiment shown is representative of three independent experiments, and the mean ± SD of three independent biological replicates are displayed in each bar. *P*-values were determined using a two-sided Student’s *t*-test. *, **, and *** indicate *P* < 0.01, *P* < 0.001, and *P* < 0.0001, respectively, compared to untreated cells. Source data are provided as a Source Data file.

**Fig. S6**

**
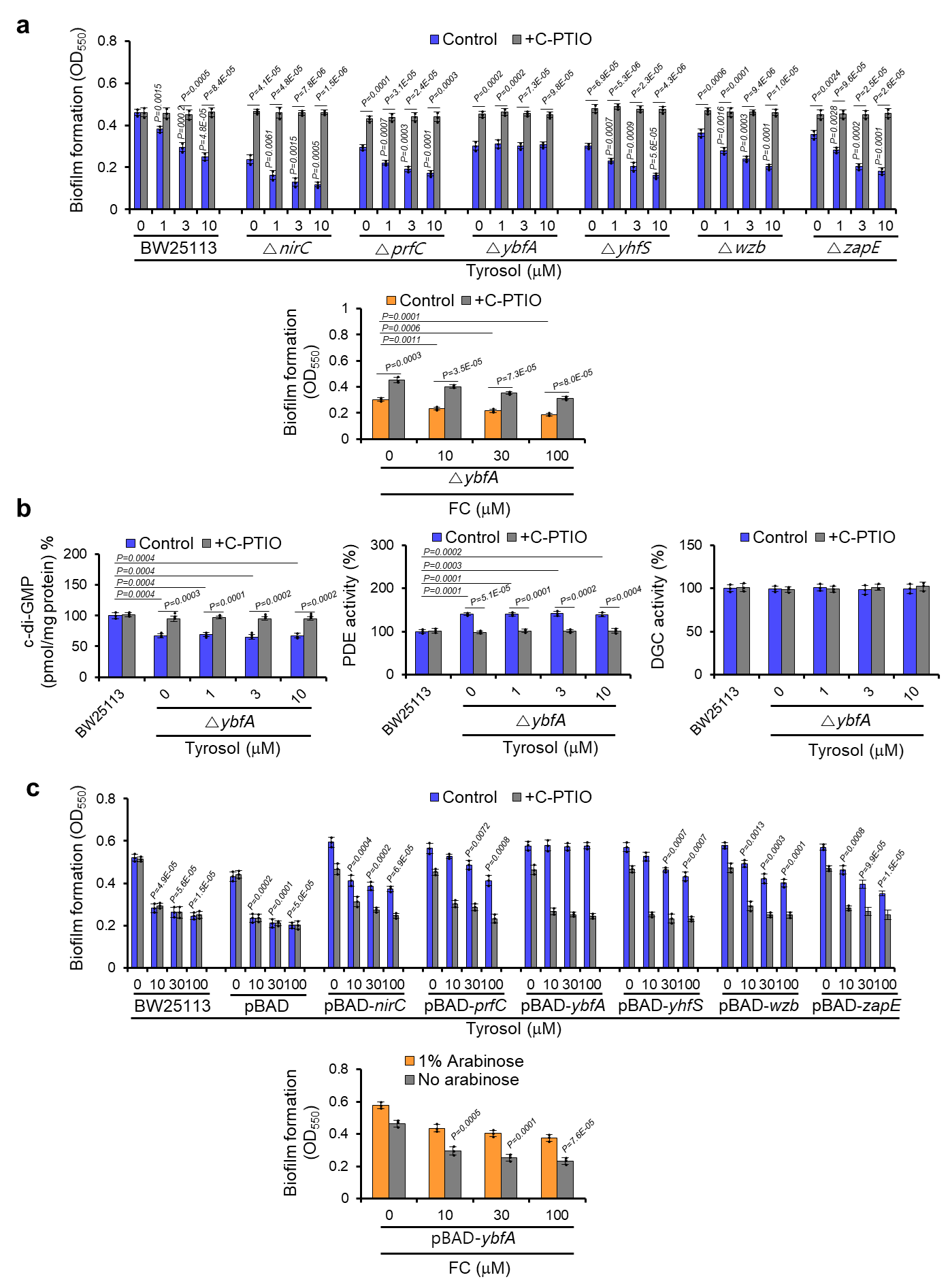
**

**Fig. S6**. Screening of *E. coli* mutants and overexpression assays result in selection of YbfA as a target of tyrosol.

(**a**) Effects of tyrosol on biofilm formation of *E. coli* mutants. The six *E. coli* mutants (Δ*yhfS*, Δ*nirC*, Δ*ybfA*, Δ*prfC,* Δ*wzb,* and Δ*zapE*) that have the same phenotype as tyrosol-treated cells screened out from the Keio mutant library were grown with different tyrosol of concentrations in the absence or presence of C-PTIO (2-(4-carboxyphenyl)-4,4,5,5-tetramethylimidazoline-1-oxyl-3-oxide) for 18 h. (**b**) Effects of tyrosol on c-di-GMP levels and PDE activity in the *ybfA* mutant. (**c**) Overexpression assays. Biofilm formation in *E. coli* BW25113, *E. coli* BW25113 containing only a vector (pBAD), and six overexpression *E. coli* BW25113 strains (pBAD-*nirC*, pBAD-*prfC*, pBAD-*ybfA*, pBAD-*yhfS*, pBAD-*wzb*, and pBAD-*zapE*) cultured in the presence of different concentrations of tyrosol and in the presence or absence of arabinose for 18 h. In **a**, **b**, and **c**, the experiment shown is representative of three independent experiments, and the mean ± SD of three independent biological replicates are displayed in each bar. *P*-values from were determined using a two-sided Student’s *t*-test and, unless otherwise indicated, data were compared with untreated cells. Source data are provided as a Source Data file.

**Fig. S7**

**
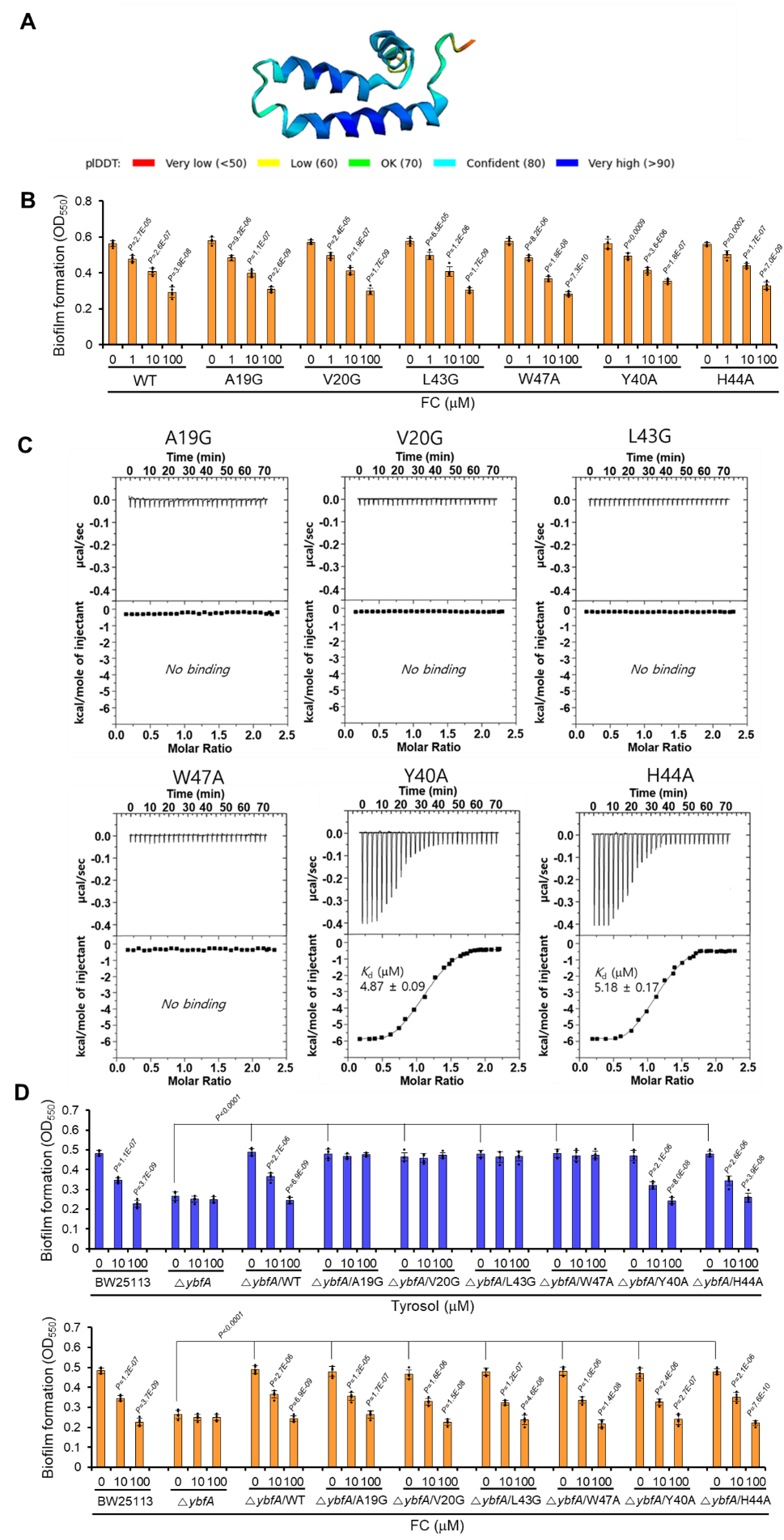
**

**Fig. S7.** Determination of key amino acid residues in YbfA interacting with tyrosol. (**a**) 3D model of YbfA. It was constructed by ColabFold, an AlphaFold2-powered and user-friendly tool. The predicted Local Distance Difference Test (pLDDT) values reflect the confidence level of each residue’s prediction. (**b**) The effects of furanone C-30 (FC), as positive control, on biofilm formation of strains overexpressing YbfA variants. Biofilm formation in *E. coli* BW25113 strains overexpressing YbfA variants was tested compared with the *E. coli* BW25113 strain overexpressing YbfA wild-type (WT) in the presence of different concentrations of FC and in the presence of arabinose for 18 h. (**c**) Measurements of the binding affinities of YbfA variants to tyrosol using ITC. The dissociation constants (*K*_d_) of YbfA variants were derived from ITC binding experiments with tyrosol. Data are presented as the mean ± SD of three independent experiments. (**d**) The effects of tyrosol, or FC as positive control, on biofilm formation of Δ*ybfA* strains complemented with YbfA variants. Biofilm formation in Δ*ybfA* strains complemented with YbfA variants were examined compared with the Δ*ybfA* strain or those complemented with YbfA WT in the presence of different concentrations of tyrosol or FC and in the presence of arabinose for 18 h. In **b** and **d**, the experiment shown is representative of three independent experiments, and the mean ± SD of five independent biological replicates are displayed in each bar. *P*-values were determined using a two-sided Student’s *t*-test and, unless otherwise indicated, data were compared with untreated cells. Source data are provided as a Source Data file.

**Fig. S8**

**
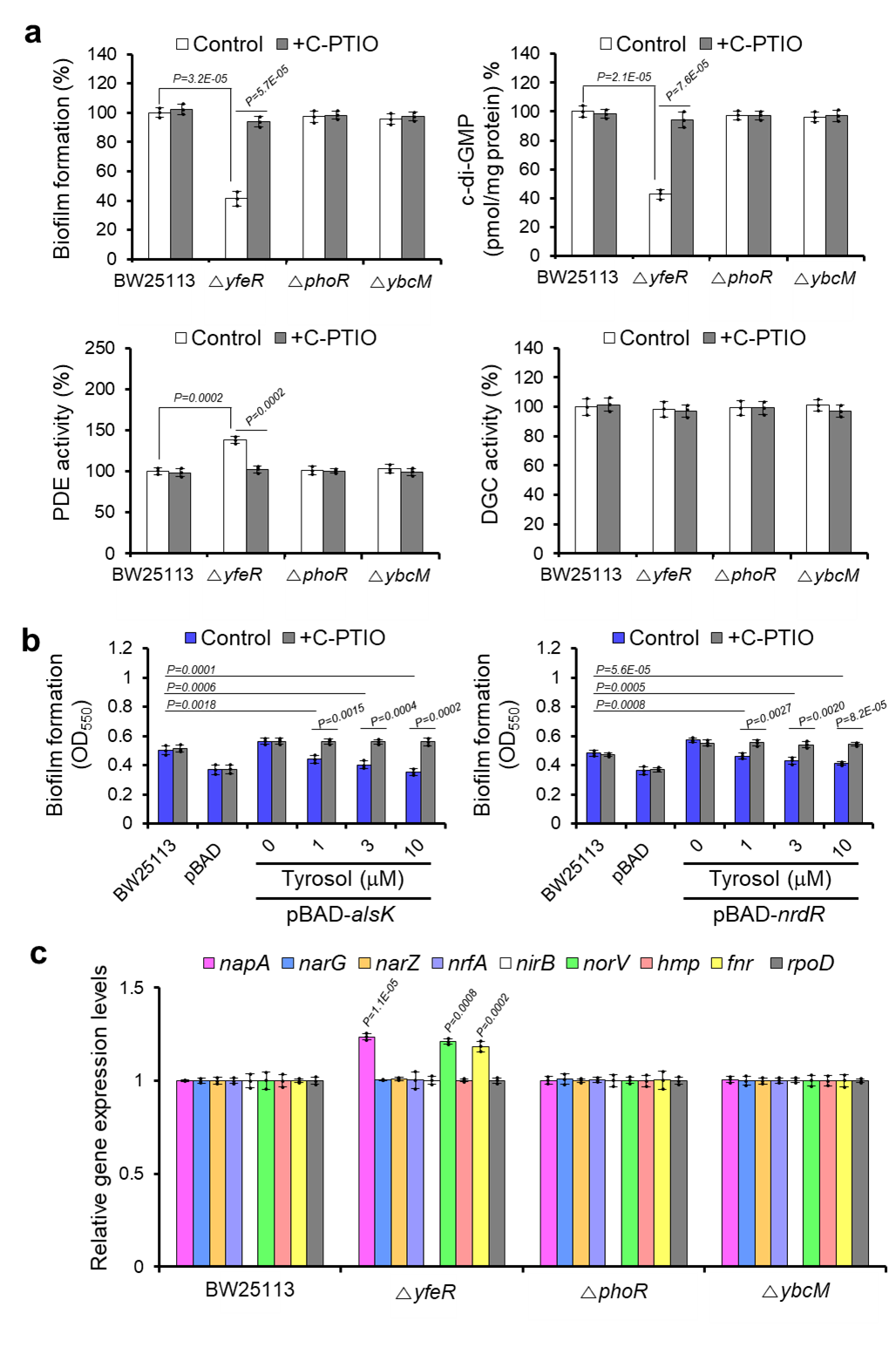
**

**Fig. S8**. Mutation or overexpression assays of five target candidate genes that was selected from differential RNA-seq analysis result in selection of YfeR as a downstream target protein of YbfA.

(**a**) The *yfeR* mutant showed the same biofilm phenotypes as the *ybfA* mutant. Biofilm formation, c-di-GMP levels, PDE, or DGC activity in three mutants (△*yfeR*, △*ybcM*, and △*phoR*). (**b**) The *alsk* or *nrdR* overexpression did not affect antibiofilm activity of tyrosol. Effects of tyrosol on biofilm formation in two overexpression strains (pBAD-*alsk* and pBAD-*nrdR*). . (**c**) The *yfeR* mutant showed the same transcription phenotypes in *napA* and *fnr* as the *ybfA* mutant. Expression of the denitrification genes and related genes in biofilms of *E. coli* mutants formed for 18 h as assessed by RT-qPCR. In **a** and **b**, the experiment shown is representative of three independent experiments, and the mean ± SD of three independent biological replicates are displayed in each bar. *P*-values were determined using a two-sided Student’s *t*-test. In **c**, the experiment shown is representative of three independent experiments, and the mean ± SD of three independent biological replicates are displayed in each bar. *P*-values were determined using a two-sided Student’s *t*-test and data were compared with wild-type *E. coli* BW25113.. Source data are provided as a Source Data file.

**Fig. S9**

**
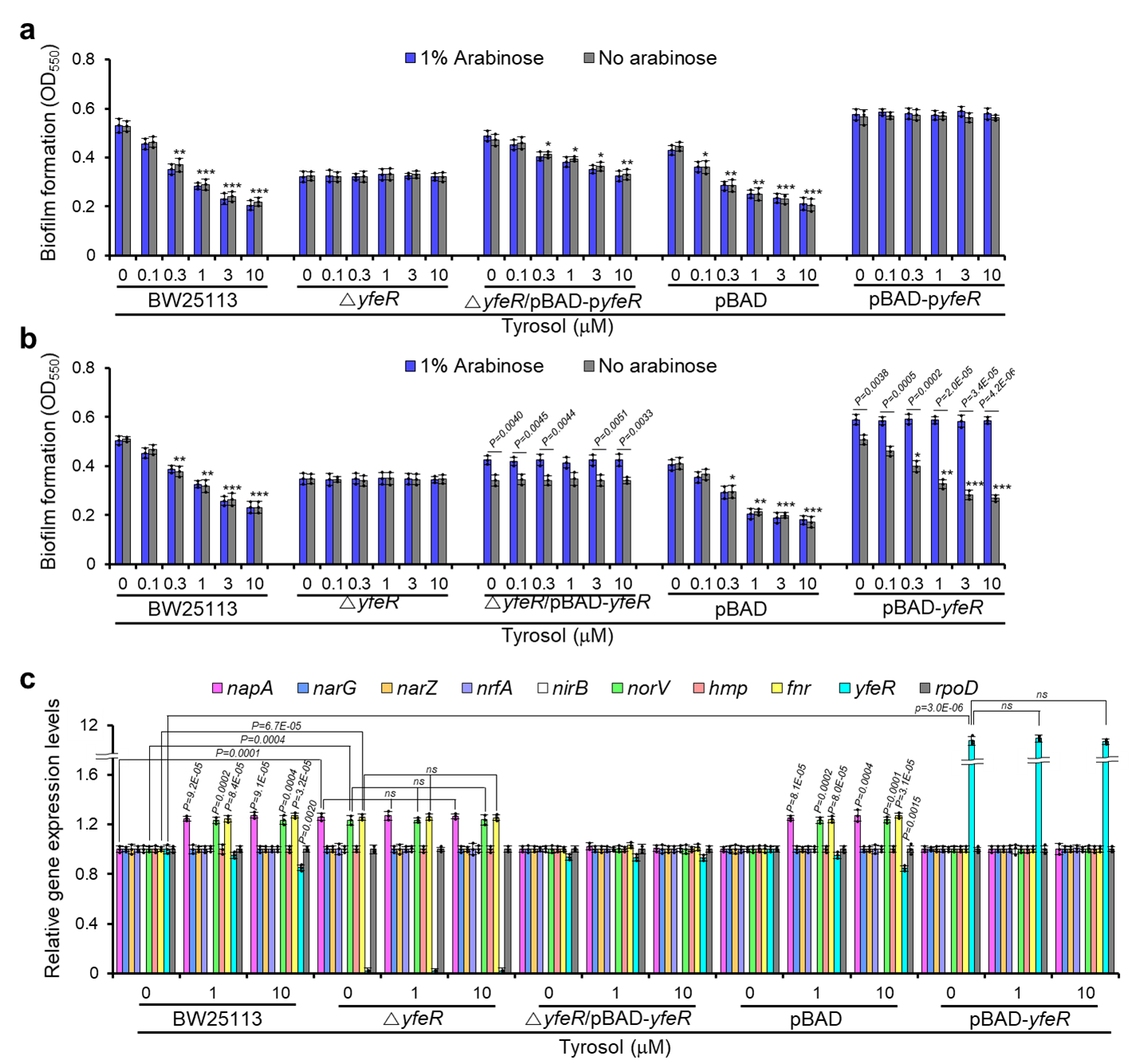
**

**Fig. S9**. Tyrosol shows antibiofilm activity by downregulating *yfeR* transcription in *E. coli*.

(**a**, **b**) Tyrosol requires the promoter region of *yfeR* for its antibiofilm activity. Biofilm formation in *E. coli* BW25113, an *yfeR* mutant (△*yfeR*), the △*yfeR* strain complemented with *yfeR* (**A**) or p*yfeR* (*yfeR* with an promoter region) (**b**) (Δ*yfeR* (*pBAD-*△*yfeR*) or Δ *yfeR* (*pBAD-*△p*yfeR*), *E. coli* BW25113 containing only a vector (pBAD), and a *yfeR* (**a**) or p*yfeR* (**b**) -overexpressing *E. coli* BW25113 strain (pBAD- *yfeR* or pBAD-p *yfeR*) cultured in the presence of different concentrations of tyrosol and in the absence or presence of arabinose. (**c**) Tyrosol did not increase *napA* transcription without its promoter region. Expression of the NAR-encoding genes and related genes in biofilm cultures of BW25113, the Δ *yfeR* strain, the Δ *yfeR* (*pBAD-*△ *yfeR*) strain, the pBAD strain, and the pBAD-*yfeR* strain grown with different tyrosol of concentrations for 18 h as assessed by RT-qPCR. In **a** and **b**, the experiment shown is representative of three independent experiments, and the mean ± SD of three independent biological replicates are displayed in each bar. *P*-values were determined using a two-sided Student’s *t*-test. *, **, and *** indicate *P* < 0.01, *P* < 0.001, and *P* < 0.0001, respectively, compared to untreated cells. In **c**, the experiment shown is representative of three independent experiments, and the mean ± SD of three independent biological replicates are displayed in each bar. *P*-values were determined using a two-sided Student’s *t*-test and, unless otherwise indicated, data were compared with untreated cells. ns indicates not significant. Source data are provided as a Source Data file.

**Fig. S10**

**
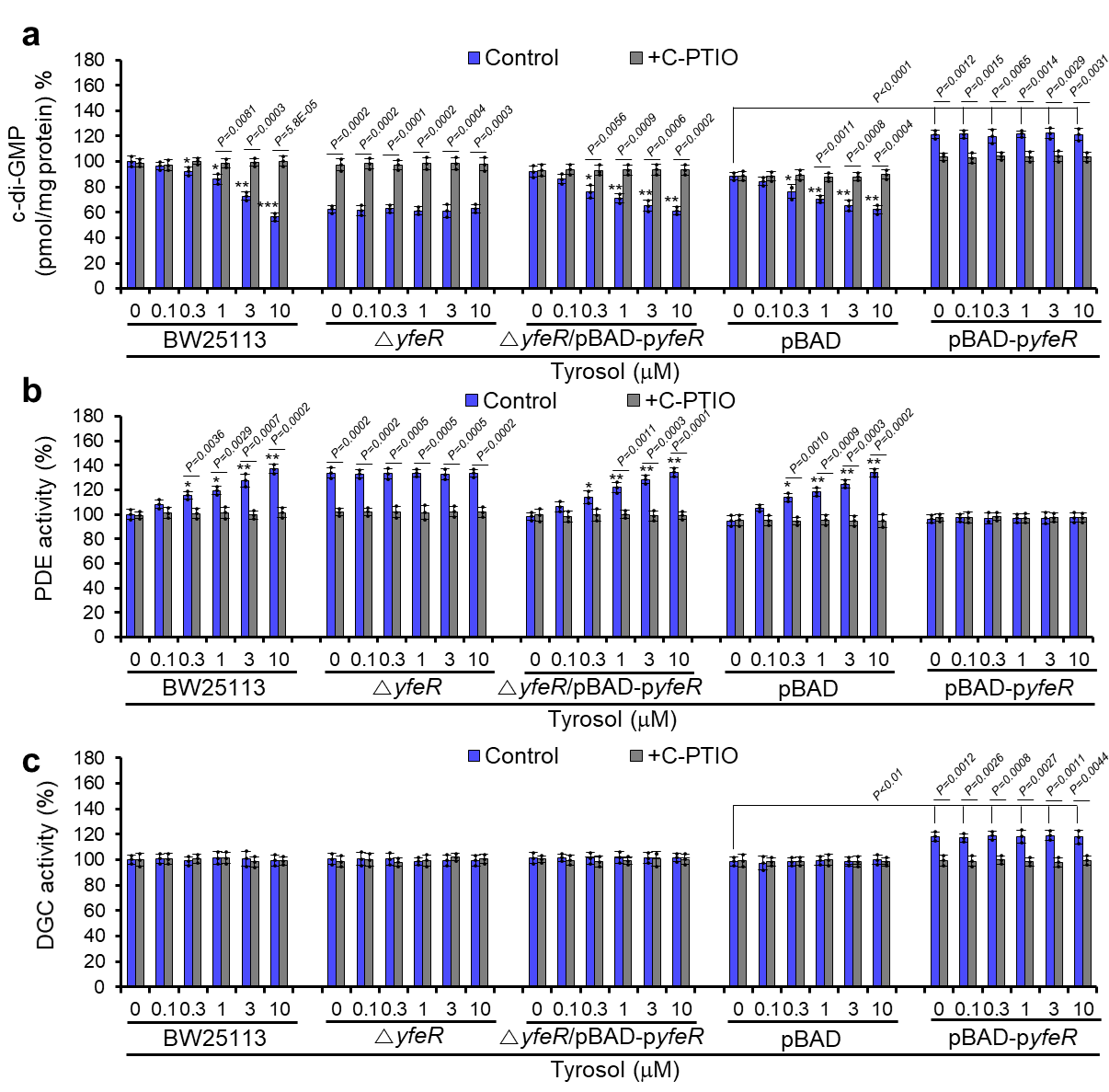
**

**Fig. S10**. Complementation and overexpression of *yfeR* containing a promoter region reverses c-di-GMP levels and PDE phenotypes induced by tyrosol. (**a, b, c**) Cellular c-di-GMP levels (**a**), PDE (**b**), and DGC (**c**) activity in *E. coli* BW25113, an *yfeR* mutant (△*yfeR*), the △*yfeR* strain complemented with p*yfeR* (*yfeR* with an promoter region) *(*Δ*yfeR* (*pBAD-*p*yfeR*)), *E. coli* BW25113 containing only a vector (pBAD), and a p*yfeR*-overexpressing *E. coli* BW25113 strain (pBAD-p*yfeR*) cultured in the presence of different concentrations of tyrosol and in the absence or presence of C-PTIO (2-(4-carboxyphenyl)-4,4,5,5-tetramethylimidazoline-1-oxyl-3-oxide). The experiment shown is representative of three independent experiments, and the mean ± SD of three independent biological replicates are displayed in each bar. *P*-values were determined using a two-sided Student’s *t*-test. *, **, and *** indicate *P* < 0.01, *P* < 0.001, and *P* < 0.0001, respectively, compared to untreated cells. Source data are provided as a Source Data file.

**Fig. S11**

**
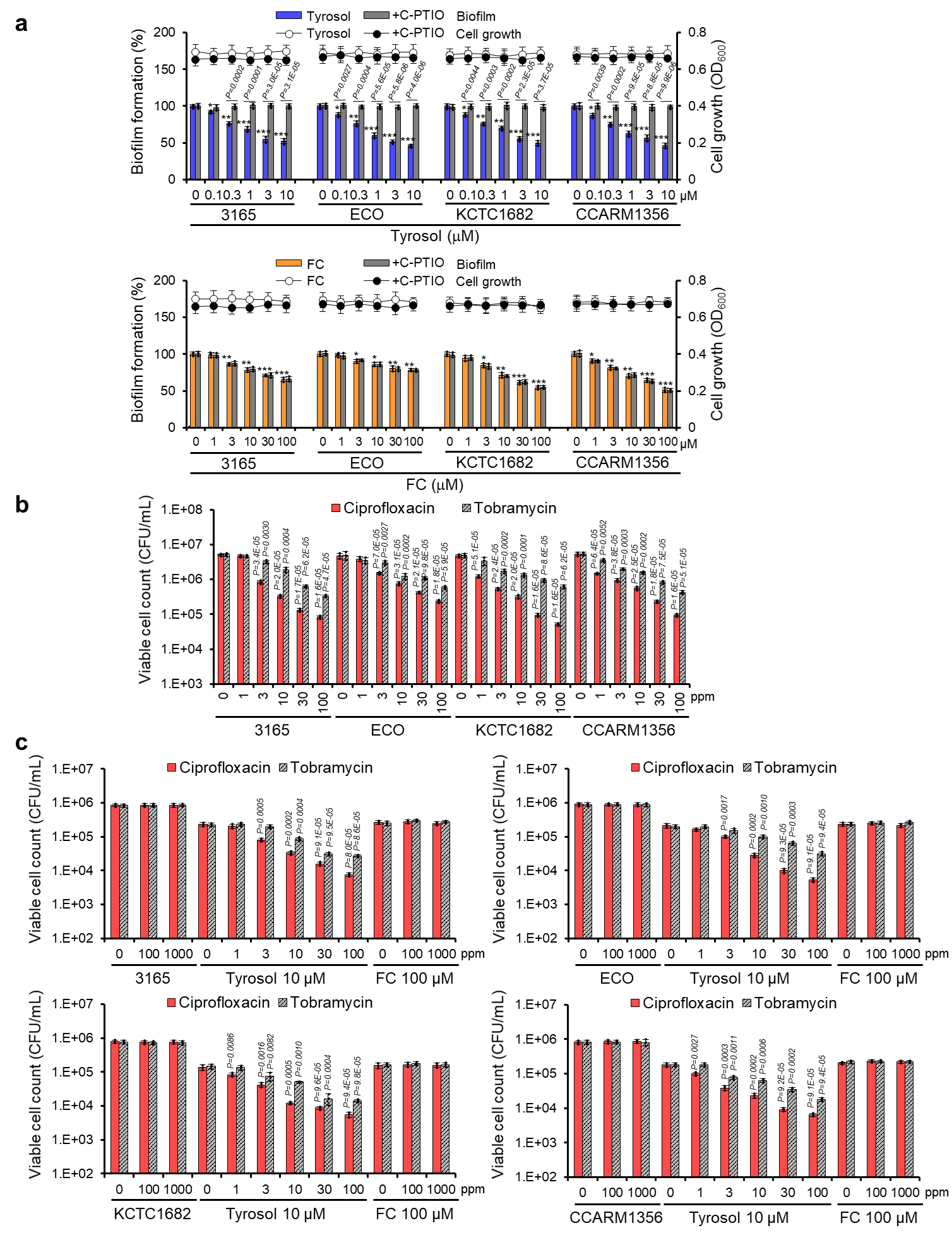
**

**Fig. S11**. Tyrosol increases the antibiotic susceptibility of biofilm cells in *E. coli* clinical isolates. (**a**) Tyrosol inhibited biofilm formation of four *E. coli* clinical isolates, including 3165, ECO, KCTC1682, and CCARM1356, without affecting cell viability, and these effects were blocked by the NO scavenger C-PTIO. (**b**) Planktonic cells of the *E. coli* clinical isolates were sensitive to ciprofloxacin and tobramycin. (**c**) Biofilm cells of the *E. coli* clinical isolates were highly resistant to ciprofloxacin and tobramycin, but tyrosol increased the antibiotic susceptibility of biofilm cells in the *E. coli* clinical isolates to ciprofloxacin and tobramycin. Furanone C-30 (FC) was used as a control. These experiments were performed in the same manner as those in *E. coli* BW25113 in Figs. 1b and 9. In **a**, the experiment shown is representative of two independent experiments, and data are presented as the mean ± SD of three independent biological replicates. *P*-values from all data were determined using a two-sided Student’s *t*-test. *, **, and *** indicate *P* < 0.01, *P* < 0.001, and *P* < 0.0001, respectively, compared to untreated cells. In **b** and **c**, the experiment shown is representative of two independent experiments, and data are presented as the mean ± SD of three independent biological replicates. *P*-values were determined using a two-sided Student’s *t*-test and data were compared to untreated cells. Source data are provided as a Source Data file.

**Fig. S12**


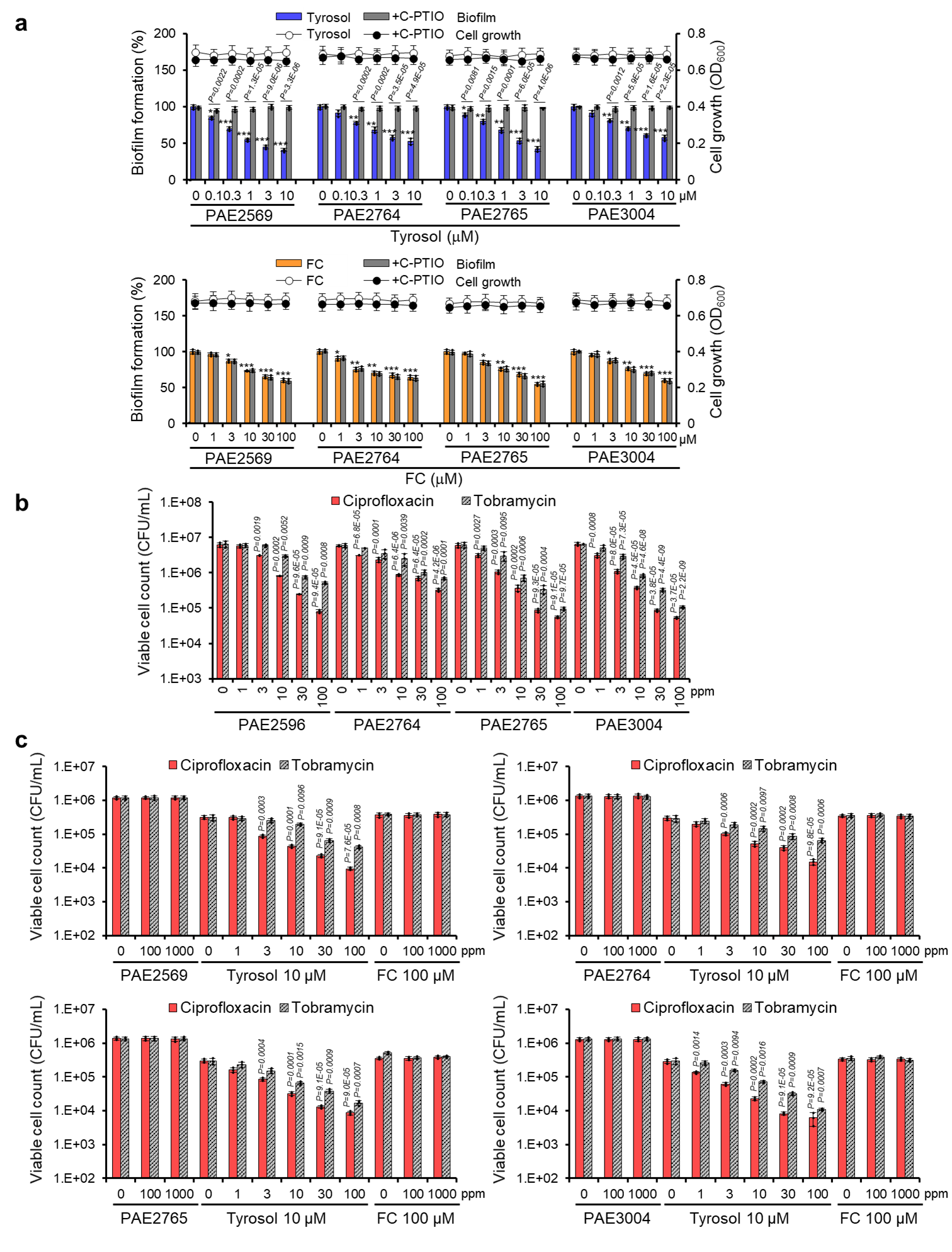


**Fig. S12**. Tyrosol increases the antibiotic susceptibility of biofilm cells in *P. aeruginosa* clinical isolates. (**a**) Tyrosol inhibited biofilm formation of four *P. aeruginosa* clinical isolates, including PAE 2569, PAE 2764, PAE 2765, and PAE 3004, without affecting cell viability, and these effects were blocked by the NO scavenger C-PTIO (2-(4-carboxyphenyl)-4,4,5,5-tetramethylimidazoline-1-oxyl-3-oxide). (**b**) Planktonic cells of the *P. aeruginosa* clinical isolates were sensitive to ciprofloxacin and tobramycin. (**c**) Biofilm cells of the *P. aeruginosa* clinical isolates were highly resistant to ciprofloxacin and tobramycin, but tyrosol increased the antibiotic susceptibility of biofilm cells in the *P. aeruginosa* clinical isolates to ciprofloxacin and tobramycin. Furanone C-30 (FC) was used as a control. These experiments were performed in the same manner as those in *P. aeruginosa* PA14 and *E. coli* BW25113 in Figs. S1bi and 9, respectively. In **a**, the experiment shown is representative of two independent experiments, and data are presented as the mean ± SD of three independent biological replicates. *P*-values were determined using a two-sided Student’s *t*-test. *, **, and *** indicate *P* < 0.01, *P* < 0.001, and *P* < 0.0001, respectively, compared to untreated cells. In **b** and **c**, the experiment shown is representative of two independent experiments, and data are presented as the mean ± SD of three independent biological replicates. *P*-values were determined using a two-sided Student’s *t*-test and data were compared to untreated cells. Source data are provided as a Source Data file.
