# Supplementary material for: Tyrosol blocks E. coli anaerobic biofilm formation via YbfA and FNR to increase antibiotic susceptibility
Source: Nat Commun. 2024 Jul 6;15:5683. doi: 10.1038/s41467-024-50116-3 (PMC11227560; doi:10.1038/s41467-024-50116-3)
Supplement: Supplementary file 3 — Description of Additional Supplementary Files [file 41467_2024_50116_MOESM3_ESM.pdf]

Description of Additional Supplementary Files

Supplementary Data 1: Genes differentially expressed in the ybfA mutant biofilm cells and their expressions in nirC or wzb mutant biofilm cells. Genes up or downregulated significantly in the ybfA mutant compared to those in both the nirC and wzb mutants are highlighted in yellow. Fold changes and p-values were extracted using a two-sided nbinomWald Test in the DESeq2 package from three biological replicates per group. Adjustments were not made for multiple comparisons.
